# Soil Organic Carbon Pool and Its Chemical Composition in *Phyllostachy pubescens* Forests at Two Altitudes in Jian-ou City, China

**DOI:** 10.1371/journal.pone.0146029

**Published:** 2015-12-30

**Authors:** Haibao Ji, Shunyao Zhuang, Zhaoliang Zhu, Zheke Zhong

**Affiliations:** 1 State Key Lab of Soil and Sustainable Agriculture, Institute of Soil Science, Chinese Academy of Sciences, Nanjing, Jiangsu Province, China; 2 University of Chinese Academy of Sciences, Beijing, China; 3 China Bamboo Research Center, Chinese Academy of Forestry, Hangzhou, Zhejiang Province, China; Institute of Tibetan Plateau Research, CHINA

## Abstract

*Phyllostachys pubescens* forests play an important role in soil organic carbon (SOC) sequestration in terrestrial ecosystems. However, the estimation and mechanism of SOC sequestration by *P*. *pubescens* forests remain unclear. In this study, the effect of *P*. *pubescens* forest distribution with elevation was investigated at two altitude sites in Jian-ou City, Southeast China. SOC storage was estimated and its chemical composition was obtained via ^13^C-nuclear magnetic resonance (NMR), chemical classification, and spectral analysis. Results showed that the SOC contents and stocks were significantly higher at the high-altitude site than at the low-altitude site in the entire soil profile (0–60 cm). The C contents of the three combined humus forms exhibited similar responses to the elevation change, and all of these forms were higher at the high-altitude site than at the low-altitude site regardless of soil layer. However, the proportions of the three combined humus C showed no significant differences between the two altitudes. The results of ^13^C-NMR showed that the SOC chemical composition did not significantly vary with elevation as well. This finding was consistent with the E_465_/E_665_ of the loosely combined humus. Overall, the results suggested that altitude should be considered during regional SOC estimation and that altitude affected the quantity rather than the quality of the SOC under the same *P*. *pubescens* vegetation.

## Introduction

The global soil organic carbon (SOC) storage is estimated at 1,550 Pg, which exceeds the cumulative pool of atmospheric C (760 Pg) and biotic C (560 Pg) [1]. Accordingly, any small change in the soil C pool size may considerably affect the atmospheric CO_2_ content [2]. Forest ecosystems contain a globally significant amount of C, i.e., approximately half of the earth’s terrestrial C (1,146 Pg), of which two-thirds (787 Pg) reside in forest soils [3, 4]. Mountain regions mostly containing forests represent heterogeneous environments that are vulnerable to climate change [5–7]. Understanding the relationship between mountain forest SOC and climate change is important. Changes in climatic variables, particularly precipitation and temperature along altitudinal gradients in mountain forest ecosystems, influence the type of vegetation and, consequently, the amount, chemical composition, and turnover of SOC [8, 9]. Therefore, SOC stocks become altered with elevation [10] as precipitation increases and temperature decreases [9, 11, 12]. However, studies on soil C stocks along elevation gradients in alpine regions revealed different relationships with altitude, including a decrease, unimodal response, or no change [6]. Djukic et al. [13] stated that SOC stocks increase with elevation in low-elevation forest sites but decrease with elevation in high-elevation grassland sites in the Austrian Limestone Alps. In the Swiss Alps, Leifeld et al. [14] reported that soil C stocks do not correlate with change in elevation. The variability in relationships across studies revealed the likely importance of local variability in climate–altitude relationships [6]. Therefore, studying the characteristics of SOC in different climate zones is essential.

Bamboo comprises approximately 1,500 species and 87 genera in the Bambusoideae subfamily worldwide [15]. China, with 500 species in 48 genera, has a highly rich bamboo flora [16]. Bamboo plantations are an indispensable part of forest ecosystems, especially in Southern China. From 1949 to 2009, the total bamboo plantation area increased from 1.3 million hectares (M ha) to 6 M ha mainly because of reforestation and afforestation on wastelands; bamboo forests account for approximately 3% of the total forest area in China [17]. *Phyllostachys pubescens* comprises the most important portion of bamboo forests, populating approximately 70% of the total bamboo forest area in China. *P*. *pubescens* grows naturally in a subtropical monsoon climate zone [18]. It grows at elevations between 10 and 1700 m above sea level (asl), but most of the areas actually inhabited are less than 800 m asl in the hills and mountains [19, 20]. However, no sufficient evidence is available to reflect the relationship between the sequestration of SOC and the elevation of *P*. *pubescens* growth because of limited studies. Studying the parameters of SOC and elevation in *P*. *pubescens* forests may provide an accurate estimation of C on a regional scale.

Presently, SOC stability is emphasized in the understanding of the impact of soil C on global climate change [21, 22]. In the past, C stability in the soil was traditionally evaluated by determining the decomposition rate of SOC through incubation experiments conducted in the laboratory [23]. Recently, ^13^C nuclear magnetic resonance (NMR) technology has been increasingly used to assess C stability by analyzing the C chemical structure of the soil [22, 24, 25]. The NMR technique enables the acquisition of information on the C chemical structure of entire soil samples without any physical or chemical fractionation; the method is suitable for characterizing the chemical structure of natural SOC pools [25, 26]. As reported, *P*. *pubescens* forests possess a high amount and potential in SOC sequestration [17, 27]. However, the stability of the sequestrated SOC in *P*. *pubescens* forests remains unknown, including the influence of the response of SOC to climate changes.

Soil humus forms are morphological patterns observed in the association between organic and mineral matter at the top of soil profiles; these forms serve as indicators of ecosystem carbon cycling [28]. Several scholars have analyzed the relationship between humus forms and SOC. For instance, Bonifacio et al. [29] suggested that humus forms reflect several mechanisms of organic matter stabilization and are clearly related to the capacity of the soil to store C in Northwestern Italy. Andreetta et al. [30] reported that humus forms hold a clear potential in evaluating the SOC status of Mediterranean forest soils. Soil humus comprises only a small portion existing in the free state; it is chiefly combined with minerals to form organic mineral complexes named combined humus [31]. Lu [32] showed that combined humus can be generally divided into three types on the basis of its variation in binding manner and tightness. These types include loosely, stably, and tightly combined humus, all of which differ in carbon-sequestration and soil fertility characteristics. Loosely, stably, and tightly combined humus are mainly composed of Al–Fe–humus complexes, Ca–humus complexes, and humin, respectively. The humification index can reflect the chemical composition of soil organic matter (SOM) [33]. Zech et al. [34] demonstrated that increased humification results in increased alkyl C, aromatic C, and carboxyl C contents and decreased O-alkyl C content.

Considered as a “bamboo hometown” in China, Jian-ou possesses the largest area (8.6 × 10^4^ ha) inhabited by *P*. *pubescens*, which distributes in all towns of this city. Bamboo growth mostly stretches from 100 m to as high as 1,200 m asl in this area. According to the local forestry bureau[35], Jian-ou has 2.8 × 10^8^ culms of bamboo forests. Bamboo plays an important role in the local socio-economy. In 2010, the economic income from the bamboo industry reached ¥5.3 billion, which accounted for 50% of the gross domestic product (GDP) of Jian-ou City [27].

Since the 1990s, numerous studies on SOC have been conducted in *P*. *pubescens* forests [17, 27, 36–38]. However, minimal information is available regarding the carbon-stock variation of *P*. *pubescens* with varying elevations. Moreover, soil C pools and their dynamics with *P*. *pubescens* on a regional scale have been studied less frequently. Therefore, the present study aimed to investigate the SOC pool, its chemical structure, and humification indices in *P*. *pubescens* forests at two altitudes in Jian-ou City, Southeast China for an improved understanding of SOC storage and turnover in *P*. *pubescens* forests.

## Materials and Methods

### Study site description

The study site was located in Jian-ou City (26°38′N–27°20′N, 117°58′E–118°57′E), northern part of Fujian Province, Southeast China. The area experiences a monsoonal subtropical climate with a mean annual temperature of 19.3°C and an average annual precipitation of 1,600–1,800 mm. The warmest and coldest months are July and January, respectively. The site has an average of 1,612 daylight hours and 286 frost-free days. Hilly landform dominates this region, which is near Wuyi Mountain, the likely center of origin of *P*. *pubescens* in China [19]. The soils in the experimental site were classified as “red soil” in the Chinese system of soil classification [39], equivalent to Ferralsols in the food and agriculture organization (FAO) soil classification system [40].

### Experimental design and soil sampling

Zhang et al. [41] divided the 0–1000 m elevation range into 10 elevation gradients of 100 m each in order to investigate the distribution area of *P*. *pubescens* forests in Jian-ou City. In their survey, they found the two elevation ranges (0–400 m and 400–1000 m) were occupied approximately 50% areas of *P*. *pubescens* forests among the 1000 m range, respectively. They also found that the bamboo mainly distributes in the range from 100–800 m. Hence, we selected the middle altitudes from the two elevation ranges as our sampling sites. Soil samples were collected from two bamboo forests at the 200 m (low-altitude site; LAS) and 761 m (high-altitude site; HAS) elevations asl. The sampling sites were homogeneously distributed in Jian-ou City, and permission to enter each site was given by Fujian Jian-ou Forestry Bureau, China. Three bamboo plots of 100 m^2^ (10 m × 10 m) areas were chosen in each altitude site. In a 2012 bamboo survey by Ji et al. (unpublished data), the average culm densities of these selected *P*. *pubescens* forests were 3,600 and 3,900 culms ha^−1^ at the two altitudes, respectively, and the average age of bamboo culms for these selected *P*. *pubescens* forests was between 1 and 2 years. Both culm density and culm age of these selected *P*. *pubescens* forests did not differ between elevations; however, the aboveground bamboo biomass increased with elevation, from 53 Mg ha^−1^ (LAS) to 73 Mg ha^−1^ (HAS).

Within each plot, soil samples were obtained from the 0–10 cm, 10–20 cm, 20–40 cm, and 40–60 cm layers from five randomly selected points. The soil samples from the five sampling points within the same layer were mixed to form a composite sample. The samples were transported to the laboratory, air-dried, and then sieved (2 mm) to homogenize the sample and remove visible roots for further analysis. During the field soil sampling, soil bulk-density samples were collected using a bulk density corer with a 200 cm^3^ volume.

There was no need of approval by Institutional Review Board (IRB) or Ethics Committee or by an Institutional Animal Care and Use Committee (IACUC) or equivalent animal ethics committee because our study was not human subject research and our object was *P*. *pubescens* forest which was a plantation but not an animal.

### Analysis of soil chemical and physical properties

Soil pH was analyzed using a pH meter in a 1:2.5 (w/v) soil/water extract. Total SOC was determined through the wet-combustion method with 133 mM K_2_Cr_2_O_7_ and concentrated H_2_SO_4_ at 220–230°C; the total nitrogen (N) in the digest was measured using a semi-micro Kjeldahl method [32]. Available phosphorus (P) and potassium (K) were determined through the HCl–NH_4_F extraction–colorimetry method and the NH_4_OAC extraction–flame photometry method, respectively. Inorganic N (NH_4_^+^–N and NO_3_^−^–N) was analyzed through KCl extraction–colorimetry. The water content was measured by obtaining 10 g of fresh soil sample from the bulk density sample and drying this sample at 105°C to a constant weight. The water content of each sample was used to calculate the bulk density on the basis of the volume and total fresh weight of the soil within each soil corer. All methods described above followed those of Lu [32].

The SOC content (C_c_) and SOC storage (C_t_) in each soil layer were calculated using the following formula [42]:

$${\text{C}}_{\text{c}}({\text{g kg}}^{-1})=0.58\times \text{SOM}$$

and

$${\text{C}}_{\text{t}}(\text{Mg }{\text{ha}}^{-1})={\text{C}}_{\text{c}}\times \text{BD}\times \text{D}\times 0.1,$$

where SOM is the soil organic matter (g kg^−1^), BD is the bulk density of the soil layer (g cm^−3^), and D is the sampling depth of the soil layer (cm). The coefficient 0.58 transforms SOM into SOC.

### Analysis of combined humus forms

Combined humus forms are classified as described by Lu [32]. The extraction procedures for the different forms are listed as follows: (1) the loosely combined humus was extracted using 0.1 M NaOH; (2) the stably combined humus was extracted using 0.1 M Na_4_P_2_O_7_ + 0.1 M NaOH mixed liquid (pH 13); and (3) the residue was considered as the tightly combined humus [32].

Both of the loosely and stably combined humus solution was measured at 465 and 665 nm using the ultraviolet spectrophotometer (Mapada UV-3100), respectively[43]. The E_465_/E_665_ ratio was calculated by dividing the absorbance of the sample at 465nm by that at 665nm. The E_465_/E_665_ ratio is related to the aromaticity and to the degree of condensation of the chain of aromatic carbons of the humic acids, and could be used as a humification index[44, 45]. Low E_465_/E_665_ ratio reflects a high degree of condensation of these structures while high ratio means presence of large quantities of aliphatic structures and low quantities of condensed aromatic structures [46]. This ratio also is inversely related to the degree of aromaticity, particle size, molecular weight, and acidity[47].

The loosely and stably combined humus C contents were measured by a liquid C/N analyzer, whereas the tightly combined humus C content was calculated by subtracting the sum of the loosely and stably combined humus C contents from the total humus C content [32].

### ^13^C-NMR analysis

Prior to ^13^C-NMR analysis, the soil samples were pretreated with HF to remove Fe^3+^ and Mn^2+^ from the soil and consequently increase the signal-to-noise ratio of the spectrum. The HF pretreatment was conducted using the methods of Mathers et al. [48] and Zhang et al. [49] as follows. Air-dried soil (5 g) was placed in a 100 mL centrifuge tube, to which 50 mL of HF solution (10% v/v) was added. The tube was capped, shaken at 120 rpm for 1 h, and then centrifuged for 10 min at 3,000 rpm. Afterward, the clear solution was discarded, and the residue was again treated with HF. This procedure was repeated eight times, with the shaking time solely varying across the different cycles (1 h shaking time for the first four, 12 h for the next three, and 24 h for the last cycle). The soil sample was then washed with distilled water to remove the residual HF. For each wash, 50 mL of distilled water was added to the soil sample in the centrifuge tube, which was shaken at 120 rpm for 10 min, centrifuged at 3,000 rpm for 10 min, and discarded of clear solution. This process was repeated four times. The washed soil sample was dried at 40°C in an oven, ground to pass through a 0.15 mm sieve, and then stored for further analysis.

The HF-treated soil samples were analyzed with a cross polarization magic-angle-spinning (CPMAS) solid-state NMR spectroscopy. The CPMAS NMR spectra were acquired through a Bruker Avance 300 MHz NMR spectrometer (Spectrospin, Rheinstetten, Germany). The measurement employed a 7 mm CPMAS detector, a frequency of 75 MHz, a magic-angle-spinning frequency of 5,000 Hz, a contact time of 2 ms, and a recycle delay time of 2.5 s. The external standard used for chemical shift determination was glycine (carboxyl at 176.4 ppm). According to literature [50, 51], each NMR spectrum was divided into the following four regions representing the different chemical environments of a ^13^C nucleus: alkyl C (0–45 ppm), O-alkyl C (45–110 ppm), aromatic C (110–160 ppm), and carbonyl C (160–200 ppm). We obtained the relative content of the different C fractions by measuring the area under the curve for each region. Two indices of organic matter stability were calculated as follows: (1) A/O-A = alkyl C / O–alkyl C [50] and (2) aromaticity = aromatic C / (alkyl C + O-alkyl C + aromatic C)[51].

### Statistical analysis

Data were presented as the average of triplicates. Independent t test was used to assess the effects of altitude change on each soil layer. Each elevation gradient was treated as a block, and the statistical significance of the differences in effects of the elevation change on the nutrient contents, different C forms, humification indices, and the C chemical structure of the soil was determined. An alpha level of 0.05 for significance determination was used in all statistical analyses. Statistical analyses were performed using the SPSS software version 20 (IBM, Chicago, IL, USA).

## Results

### Soil chemical and physical properties

In the 0–10 cm and 10–20 cm soil layers, the pH was higher in the HAS than in the LAS (*P* < 0.05), but no significant difference in the other layers was found (Table 1). In the profile of the LAS, soil pH increased with increasing depth from 4.26 to 4.58. However, no significant difference in soil pH across different soil depths was observed in the profile of the HAS. The SOC contents decreased with increasing soil depths from 19.04 g kg^−1^ to 9.72 g kg^−1^ and from 34.37 g kg^−1^ to 12.41 g kg^−1^ in the LAS and HAS, respectively. In general, the SOC content was higher in the HAS than in the LAS. The soil total nitrogen exhibited a distribution pattern similar to that of the SOC. However, inorganic nitrogen, available phosphorus, available potassium, and bulk density were higher at the LAS than at the HAS (*P* > 0.05). All of these indicators were closely related to the soil depth. The soil inorganic nitrogen as well as the available phosphorus and potassium decreased while the soil bulk density increased with increasing soil depth (Table 1).

**Table 1:** Selected soil chemical and physical properties in *Phyllostachys pubescens* forests at two altitudes^a^.

Soil profile	Elevation	pH	Organic C (g kg^−1^)	Total N (g kg^−1^)	Inorganic N (mg kg^−1^)	Available P (mg kg^−1^)	Available K (mg kg^−1^)	Bulk density (g cm^−3^)
**0–10 cm**	**LAS**	4.26 b	19.04 b	1.16 b	42.29 a	15.91 a	59.00 a	0.90 a
	**HAS**	4.66 a	34.37 a	1.94 a	29.60 a	10.29 a	55.00 a	0.75 a
**10–20 cm**	**LAS**	4.32 b	16.84 b	0.79 b	35.24 a	11.14 a	49.33 a	0.91 a
	**HAS**	4.65 a	27.19 a	1.73 a	25.01 a	7.16 a	39.67 a	0.83 a
**20–40 cm**	**LAS**	4.46 a	12.73 b	0.91 b	25.44 a	8.43 a	38.00 a	1.11 a
	**HAS**	4.62 a	22.09 a	1.65 a	24.07 a	5.45 a	35.67 a	0.98 a
**40–60 cm**	**LAS**	4.58 a	9.72 a	0.79 a	18.36 a	5.93 a	26.33 a	1.08 a
	**HAS**	4.66 a	12.41 a	0.85 a	15.58 a	3.24 b	20.67 a	1.12 a

LAS: low-altitude site; HAS: high-altitude site.

^a^ Means with different letters indicate significant differences between the two altitudes for each parameter within each soil layer at *P* = 0.05 level according to the independent t test.

### SOC storage

**Fig 1:**
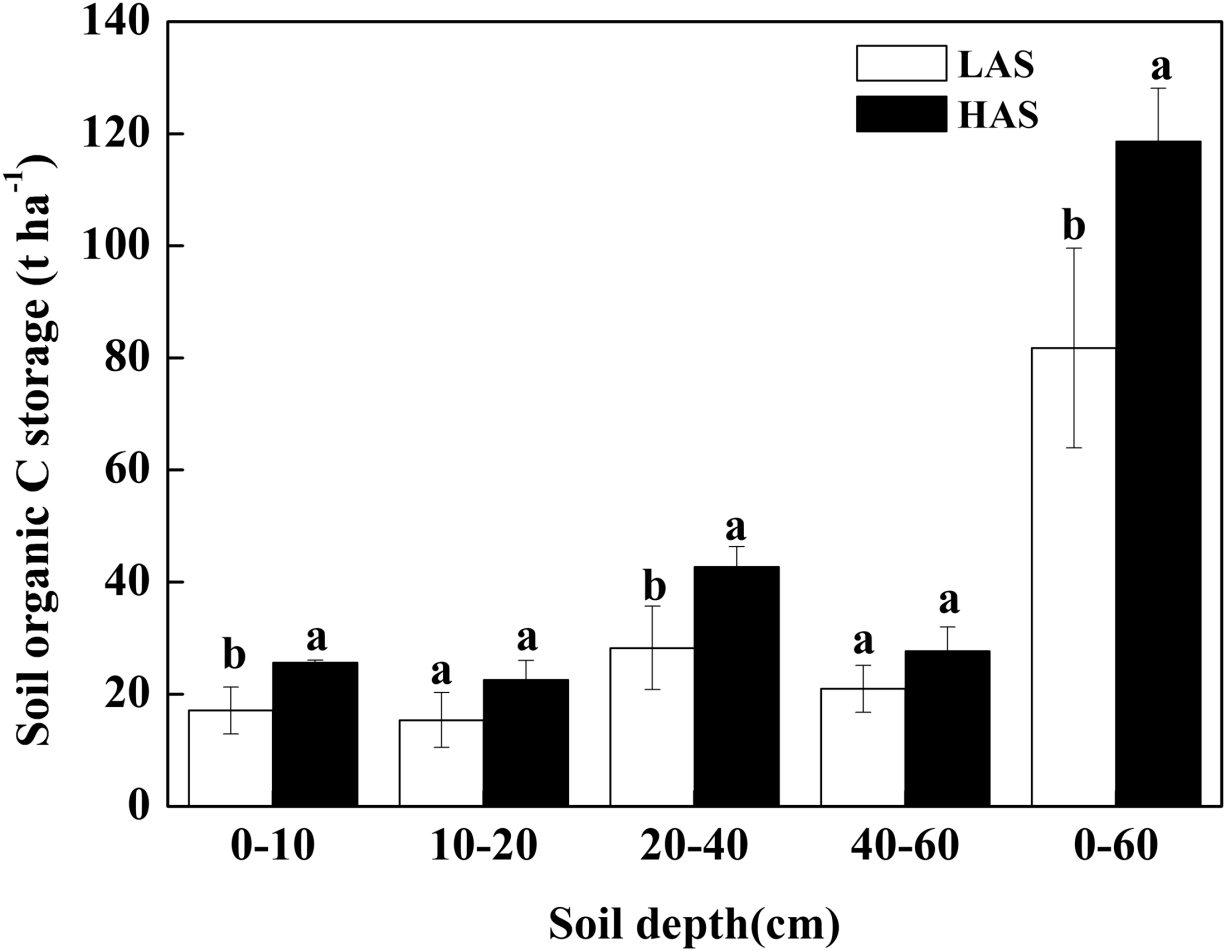
Comparison of SOC storage in the different soil layers between two elevations. LAS: low-altitude site; HAS: high-altitude site.

As shown in Fig 1, the SOC storage in the 0–60 cm layer amounted to 81.75 Mg hm^−2^ in the LAS, which was lower than 118.6 Mg hm^−2^ in the HAS (*P* < 0.05). In the 0–10 cm, 10–20 cm, 20–40 cm, and 40–60 cm layers, SOC storage was also lower in the LAS than in the HAS. However, the difference in SOC content between the two sites was significant only in the 0–10 cm and 20–40 cm layers and not in the 10–20 cm and 40–60 cm layers(*P* < 0.05).

### Soil combined humus carbon forms

**Fig 2:**
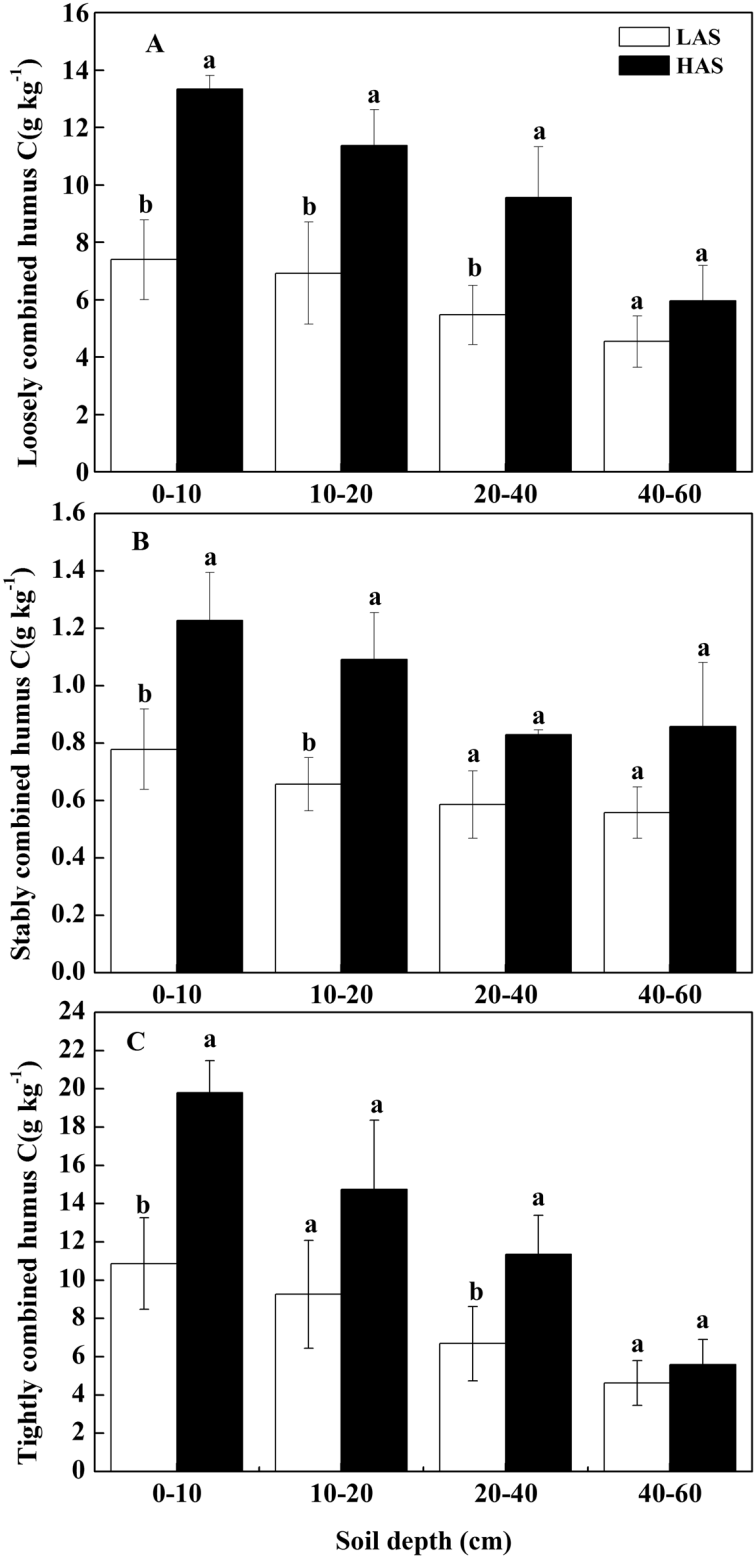
Comparison of the C contents of combined humus forms in the different soil layers between two elevations. C contents of (A) loosely combined humus, (B) stably combined humus, and (C) tightly combined humus. LAS: low-altitude site; HAS: high-altitude site.

The three types of combined humus C (loosely, stably, and tightly combined humus C) are shown in Fig 2 and Table 2. The C contents of the loosely, stably, and tightly combined humus ranged from 4.5 g kg^−1^ to 13.3 g kg^−1^ (Fig 2A), 0.6 g kg^−1^ to 1.2 g kg^−1^ (Fig 2B), and 4.6 g kg^−1^ to 19.8 g kg^−1^ (Fig 2C), respectively. In the 0–10 cm, 10–20 cm, 20–40 cm, and 40–60 cm soil layers, the C content of the loosely combined humus was 80.3%, 64.2%, 74.9%, and 31.2% higher at the HAS than at the LAS, respectively (Fig 2A). Similarly, the C content of the stably combined humus was 57.7%, 66.3%, 41.5%, and 53.9% higher (Fig 2B), and the C content of the tightly combined humus was 82.3%, 59.0%, 69.9%, and 20.9% higher (Fig 2C) at the HAS than at the LAS in the respective soil layers. The combined humus forms were arranged on the basis of C content in the following order: tightly > loosely > stably combined humus C (Fig 2). All of the combined-humus C contents decreased with increasing soil depth at both sites (Fig 2). However, the proportions of the three combined humus C showed no significant differences between the two altitudes regardless of soil layer (*P* > 0.05) (Table 2).

**Table 2:** Proportions of combined humus C in *Phyllostachys pubescens* forests at two altitudes^a^.

		Soil Organic C (%)		
Soil profile	Elevation	Loosely combined humus C	Stably combined humus C	Tightly combined humus C
**0–10 cm**	**LAS**	38.96 a	4.12 a	56.92 a
	**HAS**	38.86 a	3.56 a	57.58 a
**10–20 cm**	**LAS**	41.36 a	4.01 a	54.64 a
	**HAS**	42.25 a	4.04 a	53.71 a
**20–40 cm**	**LAS**	43.41 a	4.63 a	51.96 a
	**HAS**	43.36 a	5.12 a	51.53 a
**40–60 cm**	**LAS**	46.86 a	5.81 a	47.33 a
	**HAS**	47.96 a	7.21 a	44.84 a

LAS: low-altitude site; HAS: high-altitude site.

^a^ Means with different letters indicate significant differences between the two altitudes for each parameter within each soil layer at *P* = 0.05 level according to the independent t test.

**Fig 3:**
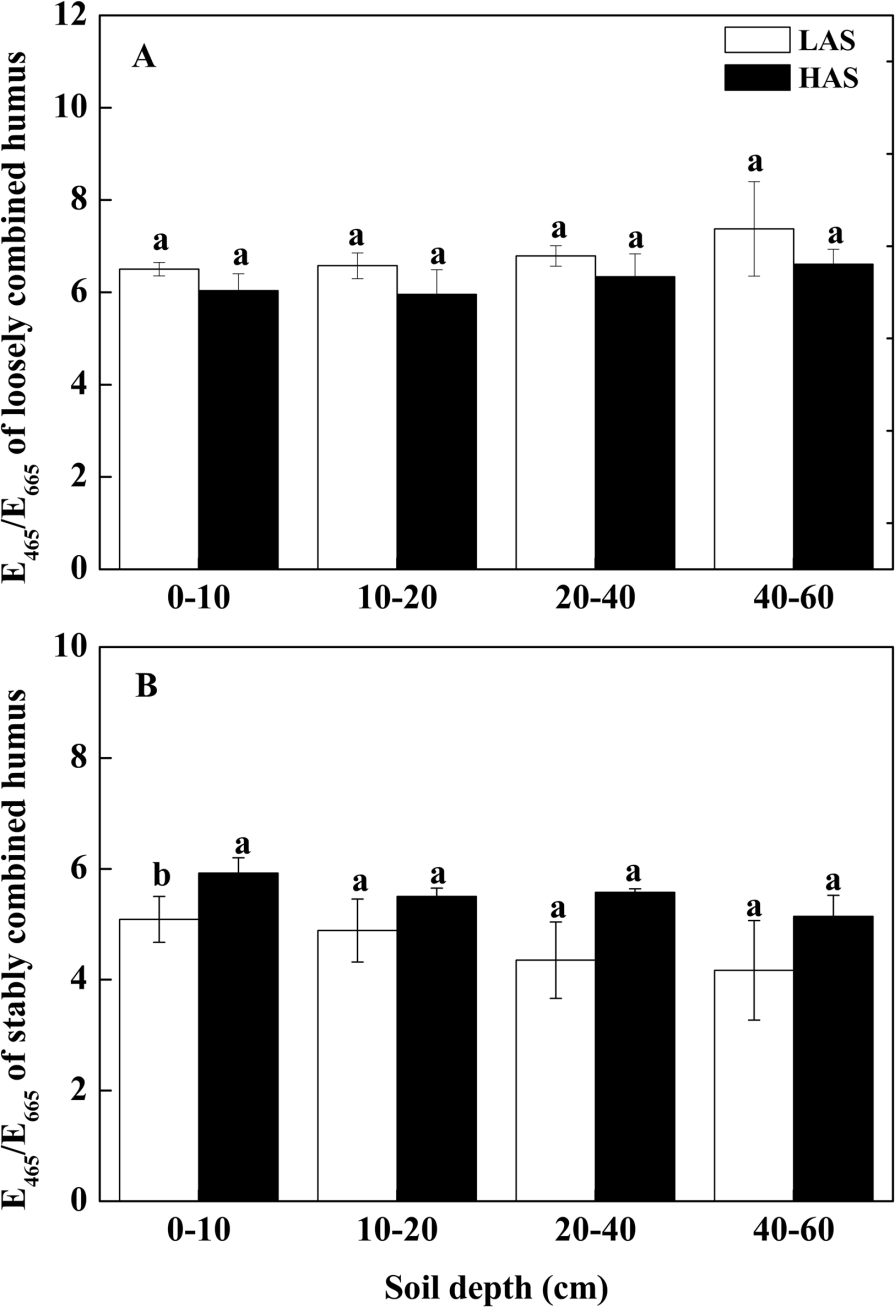
E_465_/E_665_ ratios of combined humus. (A) Loosely combined humus. (B) Stably combined humus. LAS: low-altitude site; HAS: high-altitude site.

The E_465_/E_665_ values of the loosely combined humus were higher at the LAS than at the HAS (*P* > 0.05) (Fig 3A). By contrast, those of the stably combined humus were higher at the HAS than at the LAS (*P* > 0.05) (Fig 3B).

### Chemical composition of SOC

**Fig 4:**
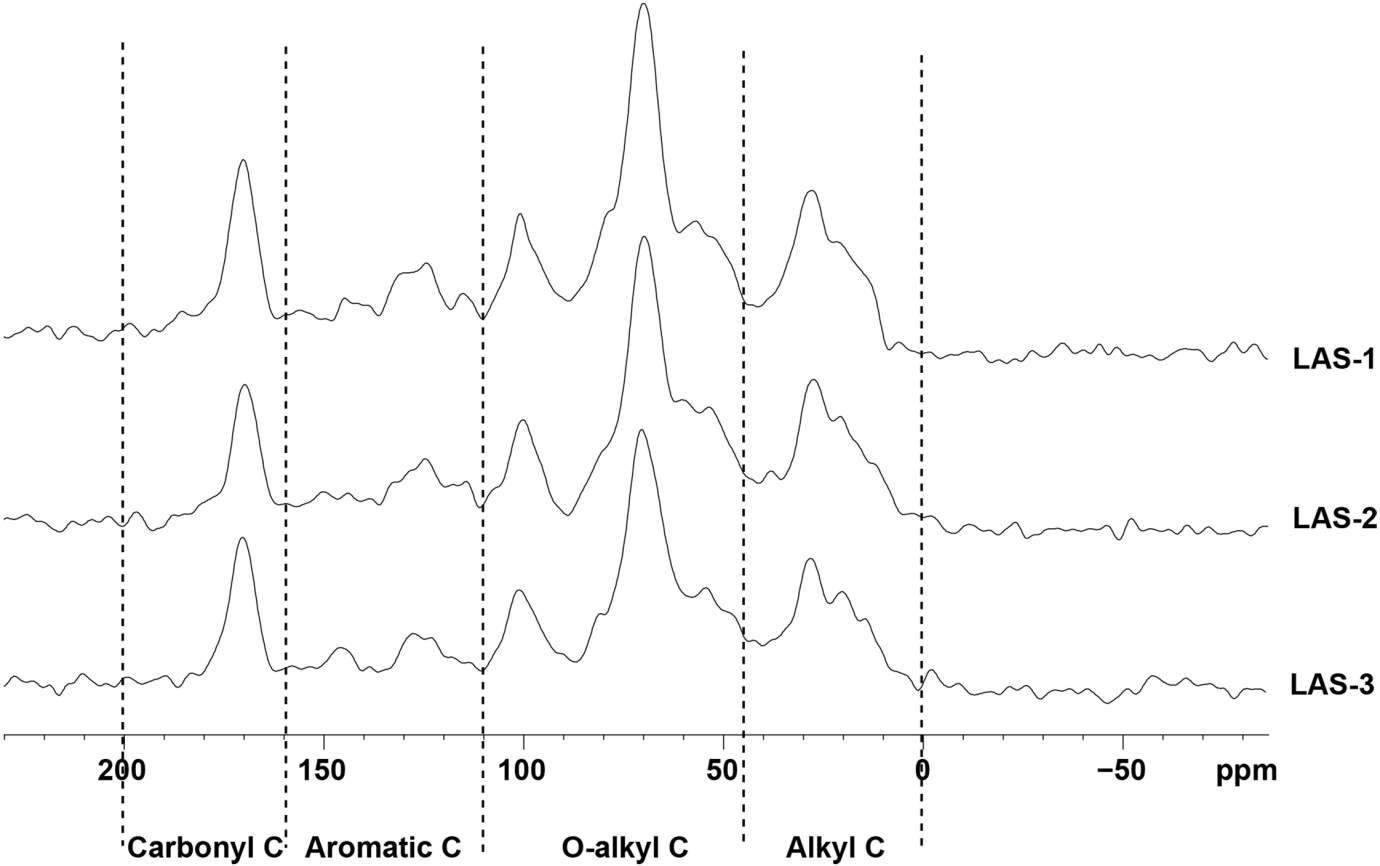
Solid-state ^13^C-NMR spectra of soil samples from the LAS. LAS: low-altitude site; HAS: high-altitude site.

**Fig 5:**
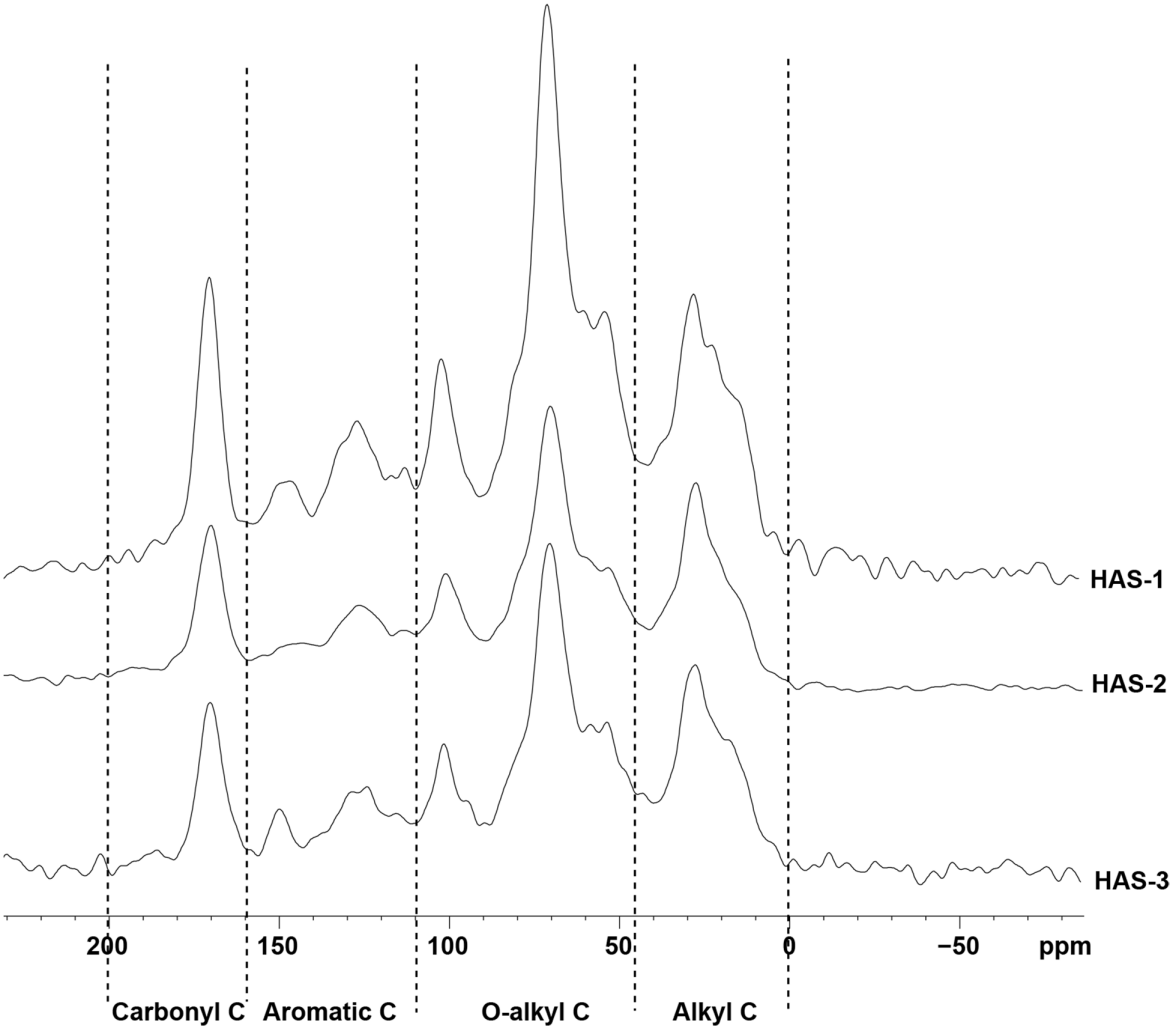
Solid-state ^13^C-NMR spectra of soil samples from the HAS. LAS: low-altitude site; HAS: high-altitude site.

The solid-state ^13^C CPMAS NMR spectra of soils in the 0–10 cm layer from the LAS and HAS are shown in Figs 4 and 5. The spectra shared similar patterns but differed in the relative intensity of the different chemical shift regions. The soil alkyl C, aromatic C content, alkyl to O-alkyl C ratio (A/O-A), and aromaticity were higher at the HAS than at the LAS, but no significant difference was found between the two sites (*P* > 0.05) (Table 3). However, the soil O-alkyl C content was significantly greater at the LAS than at the HAS (*P* < 0.05).

**Table 3:** Distribution of soil organic carbon groups based on ^13^C-NMR spectra in *Phyllostachys pubescens* forests with elevation ^a^.

Elevation	Alkyl C (%)	O-alkyl C	Aromatic C	Carbonyl C	A/O-A	Aromaticity
**LAS**	21.80 a	50.22 a	14.44 a	13.54 a	0.43 a	0.17 a
**HAS**	24.58 a	47.60 b	15.79 a	12.03 a	0.52 a	0.18 a

LAS: low-altitude site; HAS: high-altitude site.

^a^ Means with different letters indicate significant differences between the two altitudes for each parameter at the *P* = 0.05 level according to the independent t test.

## Discussion

### Effect of altitude on soil properties

Soil pH is a function of parent material, weathering time, vegetation, and climate [52]. The weathering times and vegetation types of the two sites were similar; hence, the differences in soil pH could have resulted from the decreased nitrification in the higher elevation as shown by the low nitrate levels in the HAS (S1 Table). However, Dahlgren et al. [53] reported that soil pH decreases in an oak woodland-conifer forest elevational transect in the western US, and Smith et al. [52] showed that soil pH decreases over an elevation gradient in a shrub-steppe ecosystem located in the eastern Washington State of the US. The discrepancies among different studies may be attributed to the variation in soil type, plant species, local climate, and other factors.

The obtained nitrogen contents showed a significant difference in the surface layers between the two altitude sites (Table 1). This result is consistent with the reports on the different types of mountain ecosystems [54, 55]. Low temperature and high moisture may have instigated such a result. However, the inorganic N, available P, and available K contents were higher at the LAS than at the HAS regardless of soil layer (*P* > 0.05), suggesting the decreased soil microbial activities in some extent.

### Effect of altitude on soil C storage and pools

The increase in SOC with increasing elevation had been previously reported in different types of mountain ecosystems [52, 56–58]. In the present study, the HAS contained higher SOC than the LAS in the 0–40 cm layers (Table 1). This SOC distribution pattern in the soil profile is expected because the underground portion of the *P*. *pubescens* plant is mainly distributed in the 0–30 cm layer, in which a greater amount of SOC secreted from this part is retained by the subsoil.

Incorporated with the bulk density, the SOC storage in the 0–60 cm layers was 45% higher in the HAS than in the LAS (Fig 1). Many researchers also reported an increase in SOC stocks with increasing altitude [59–64]. This increase likely originates from two sources as follows: (1) greater bamboo biomass along the elevation gradients and (2) decreasing litter decomposition with increasing altitude. The increase in tree biomass with increasing altitude indicates greater C input to the soil at high elevations. The result was comparable with a similar study performed in bamboo plantations, in which bamboo harvest reduced and litterfall input increased consequently with increasing altitude [65]. Smith et al. [52] found that the total soil C in a semi-arid shrub-steppe ecosystem increases with elevation probably because of the larger amount of plant biomass that results from greater precipitation at higher elevations. In general, temperature decreases with increasing elevation [10, 66, 67]. Low temperatures at high altitudes are useful in maintaining a low SOM decomposition rate [64]. Slowed litter decomposition and soil N mineralization at high elevations could presumably lead to the accumulation of total C content in the soils [54, 66], as we observed in the *P*. *pubescens* forests of the HAS. Therefore, elevation exerted a considerable influence on the SOC storage of *P*. *pubescens* forests. This altitude effect should be considered in future regional SOC storage estimation and management.

### Effect of altitude on SOC chemical composition

In this study, the different combined humus C contents showed similar responses to elevation change (Fig 2). The three combined humus C contents in all layers increased with elevation, similar to the SOC. Previous studies demonstrated that a relationship exists between SOC and organo-mineral complexes. In particular, Matus et al. [68] established a significant positive relationship between SOC and Al-complexed SOC in volcanic soils of Chile. Rasmussen et al. [69] found a highly significant positive correlation between Al–humus complexes and total C content in a California conifer forest, which could reflect the site’s soil C dynamics and turnover. Our results showed that tightly combined humus C dominated the SOC in the soils of the three combined humus forms (Fig 2); this finding is consistent with the results of Ma et al. [70]. By contrast, Liu et al. [71] found that loosely combined humus C dominates the SOC in soils. The discrepancy among different studies could be caused by the differences in soil type, plant species, and other factors. However, the proportions of the three combined humus C showed no significant differences between the two altitudes (Table 2), suggesting that the three combined humus C structure was stable with the altitude in some extent.

The ^13^C-NMR technique has been used to assess the chemical characteristics of SOC [24]. Our results showed that O-alkyl C chiefly contributed to the SOC in the two altitude sites (Table 3); this finding is consistent with the results of Du et al. [58] and Zhang et al. [49]. By contrast, Jien et al. [72] found that alkyl C dominates the SOC in the soils of natural broad-leaved forests and adjacent coniferous plantations. The inconsistency among different studies could be caused by the differences in plant species, local climate, and management practices [73, 74].

In this study, we found that the alkyl C, aromatic C, and carbonyl C contents as well as the alkyl C to O-alkyl C ratio (A/O-A) and aromaticity of organic matter demonstrated no significant differences with elevation (*P* > 0.05) (Table 3). This finding is inconsistent with the results of Du et al. [58]. This discrepancy may be attributed to the differences in soil type because the soil type in our study did not differ in both altitudes evaluated. Some have argued that SOC stability is mainly dependent on soil type [75, 76] because of the minimal difference in soil chemical structure revealed by NMR spectroscopy, such as in different land-use types. In the present study, the O-alkyl C content was higher at the LAS than at the HAS (Table 3). This result implied that easily decomposed components, including polysaccharides and cellulose, enriched the LAS soil to a greater extent than the HAS soil [72].

The optical characteristics of humic substances were evaluated through the E_465_/E_665_ ratios, which provided rough estimations of the molecular dimensions, which decreased with increasing aggregated size of humic substances and degree of humification [72]. A low E_465_/E_665_ value indicates a high degree of humification [77, 78]. The E_465_/E_665_ of the loosely combined humus was higher at the LAS than at the HAS regardless of soil layer, but the difference was not significant (*P* > 0.05) (Fig 3A). Hence, the degree of condensation of the aromatic network and humification of loosely combined humus did not significantly differ. This result was consistent with that of the ^13^C CPMAS NMR spectra showing no significant difference in aromatic C content and aromaticity with the different elevations (0–10 cm layer) (Table 3). However, although the E_465_/E_665_ of stably combined humus increased from the LAS to the HAS in all soil layers, the differences were not significant (*P* > 0.05) (Fig 3B). The sampling sites contain acidic soil; hence, the loosely combined humus mainly comprised the soil profiles. Accordingly, the E_465_/E_665_ of loosely combined humus is considered a more suitable index rather than that of the stably combined humus for identifying the aromaticity and humification degree of SOC.

### Effect of altitude on SOC dynamics and turnover in *P*. *pubescens* forests

Multiple environmental factors, namely, temperature, precipitation, N deposition, litter quality, and soil type, vary with altitude; each factor can potentially affect soil C sequestration and turnover [10]. Moisture plays an important role in the growth of *P*. *pubescens* and is the limiting factor for its productivity. Therefore, the greater precipitation at the higher elevation was associated with the larger amounts of bamboo biomass. Numerous studies indicated that the decomposition of labile SOM [79, 80] and soil respiration [81, 82] increase with temperature. The mean annual temperature shows a significant negative correlation with elevation [10, 65]. However, the SOC composition did not significantly differ between the two sites, as indicated by the proportions of the three combined humus C, the ^13^C CPMAS NMR and E_465_/E_665_ results of the combined humus. Therefore, the high SOC sequestration in the *P*. *pubescens* forests of the HAS mainly resulted from the high precipitation, low temperature, and soil respiration associated with the high altitude.

## Conclusions

The SOC contents and stocks were significantly higher at the HAS than at the LAS in the entire soil profile (0–60 cm). The significant discrepancy implied that altitude should be considered in the regional C storage estimation of *P*. *pubescens* forests. The C contents of the three combined humus forms demonstrated similar responses to the elevation change in terms of C content; all of the forms showed higher C contents at the HAS than at the LAS regardless of soil layer. However, the proportions of the three combined humus C showed no significant differences between the two altitudes. The ^13^C CPMAS NMR results also showed that the SOC chemical composition did not significantly vary with elevation. This finding is consistent with the E_465_/E_665_ values of the loosely combined humus. This result suggested that the SOC exhibited a similar pattern of chemical composition when covered by the same *P*. *pubescens* vegetation.

## Supporting Information

S1 Table The content of NO_3_^−^–N in *Phyllostachys pubescens* forests at two altitudes. LAS: low-altitude site; HAS: high-altitude site.
(DOC)

## References

[1] Lal R. Soil carbon sequestration impacts on global climate change and food security. science. 2004;304(5677):1623–7. 15192216 10.1126/science.1097396

[2] Don A, Schumacher J, Freibauer A. Impact of tropical land-use change on soil organic carbon stocks—a meta-analysis. Global Change Biology. 2011;17(4):1658–70. 10.1111/j.1365-2486.2010.02336.x

[3] Dixon RK, Solomon AM, Brown S, Houghton RA, Trexier MC, Wisniewski J. Carbon pools and flux of global forest ecosystems. Science. 1994;263(5144):185–90. 17839174 10.1126/science.263.5144.185

[4] Johnson DW, Curtis PS. Effects of forest management on soil C and N storage: meta analysis. Forest Ecology and Management. 2001;140(2):227–38.

[5] Beniston M. Climatic change in mountain regions: a review of possible impacts Climate Variability and Change in High Elevation Regions: Past, Present & Future: Springer; 2003 p. 5–31.

[6] Britton AJ, Helliwell RC, Lilly A, Dawson L, Fisher JM, Coull M, et al An integrated assessment of ecosystem carbon pools and fluxes across an oceanic alpine toposequence. Plant and Soil. 2011;345(1–2):287–302. 10.1007/s11104-011-0781-3

[7] Hoffmann U, Hoffmann T, Jurasinski G, Glatzel S, Kuhn NJ. Assessing the spatial variability of soil organic carbon stocks in an alpine setting (Grindelwald, Swiss Alps). Geoderma. 2014;232–234:270–83. 10.1016/j.geoderma.2014.04.038

[8] Jobbágy EG, Jackson RB. The vertical distribution of soil organic carbon and its relation to climate and vegetation. Ecological applications. 2000;10(2):423–36.

[9] Lemenih M, Itanna F. Soil carbon stocks and turnovers in various vegetation types and arable lands along an elevation gradient in southern Ethiopia. Geoderma. 2004;123(1):177–88.

[10] Garten CT, Hanson PJ. Measured forest soil C stocks and estimated turnover times along an elevation gradient. Geoderma. 2006;136(1):342–52.

[11] Wang SQ, Huang M, Shao XM, Mickler RA, Li KR, Ji JJ. Vertical distribution of soil organic carbon in China. Environmental management. 2004;33(1):S200–S9.

[12] Hontoria C, Saa A, Rodríguez-Murillo JC. Relationships between soil organic carbon and site characteristics in peninsular Spain. Soil Science Society of America Journal. 1999;63(3):614–21.

[13] Djukic I, Zehetner F, Tatzber M, Gerzabek MH. Soil organic-matter stocks and characteristics along an Alpine elevation gradient. Journal of Plant Nutrition and Soil Science. 2010;173(1):30–8.

[14] Leifeld J, Zimmermann M, Fuhrer J, Conen F. Storage and turnover of carbon in grassland soils along an elevation gradient in the Swiss Alps. Global Change Biology. 2009;15(3):668–79.

[15] Ohrnberger D. The bamboos of the world: annotated nomenclature and literature of the species and the higher and lower taxa: Elsevier; 1999.

[16] Chen XG, Zhang XQ, Zhang YP, Booth T, He XH. Changes of carbon stocks in bamboo stands in China during 100 years. Forest Ecology and Management. 2009;258(7):1489–96.

[17] Fu WJ, Jiang PK, Zhao KL, Zhou GM, Li YF, Wu JS, et al The carbon storage in moso bamboo plantation and its spatial variation in Anji County of southeastern China. Journal of soils and sediments. 2014;14(2):320–9.

[18] Fu JH. Chinese moso bamboo: its importance. Bamboo. 2001;22(5):5–7.

[19] Fu JH. Moso bamboo in China. ABS Magazine. 2000;21:12–7.

[20] Zhang HX, Zhuang SY, Qian HY, Wang F, Ji HB. Spatial variability of the topsoil organic carbon in the moso bamboo forests of southern china in association with soil properties. Plos One. 2015;10(3):1–17. 10.1371/journal.pone.0119175 PMC4366393 25789615

[21] Shrestha BM, Certini G, Forte C, Singh BR. Soil organic matter quality under different land uses in a mountain watershed of Nepal. Soil Science Society of America Journal. 2008;72(6):1563–9.

[22] Zhou P, Pan GX, Spaccini R, Piccolo A. Molecular changes in particulate organic matter (POM) in a typical Chinese paddy soil under different long-term fertilizer treatments. European journal of soil science. 2010;61(2):231–42.

[23] Kätterer T, Reichstein M, Andrén O, Lomander A. Temperature dependence of organic matter decomposition: a critical review using literature data analyzed with different models. Biology and fertility of soils. 1998;27(3):258–62.

[24] Mathers NJ, Mao XA, Saffigna PG, Xu ZH, Berners-Price SJ, Perera MCS. Recent advances in the application of 13C and 15N NMR spectroscopy to soil organic matter studies. Soil Research. 2000;38(4):769–87.

[25] Huang ZQ, Xu ZH, Chen CR, Boyd S. Changes in soil carbon during the establishment of a hardwood plantation in subtropical Australia. Forest Ecology and management. 2008;254(1):46–55.

[26] Schnitzer M. The in situ analysis of organic matter in soils. Canadian Journal of Soil Science. 2001;81(3):249–54.

[27] Zhuang SY, Ji HB, Zhang HX, Sun B. Carbon storage estimation of Moso bamboo (Phyllostachys pubescens) forest stands in Fujian, China. Tropical Ecology. 2015;56(3):383–91.

[28] Ponge JF. Humus forms in terrestrial ecosystems: a framework to biodiversity. Soil Biology and Biochemistry. 2003;35(7):935–45.

[29] Bonifacio E, Falsone G, Petrillo M. Humus forms, organic matter stocks and carbon fractions in forest soils of northwestern Italy. Biology and Fertility of Soils. 2011;47(5):555–66. 10.1007/s00374-011-0568-y

[30] Andreetta A, Ciampalini R, Moretti P, Vingiani S, Poggio G, Matteucci G, et al Forest humus forms as potential indicators of soil carbon storage in Mediterranean environments. Biology and Fertility of Soils. 2011;47(1):31–40.

[31] Chen H, Wang Q. The behaviour of organic matter in the process of soft soil stabilization using cement. Bulletin of Engineering Geology and the Environment. 2006;65(4):445–8.

[32] Lu RK. Analytical methods of soil agrochemistry. Beijing: China Agricultural Science and Technology Press 2000.(in Chinese).

[33] Schöning I, Kögel-Knabner I. Chemical composition of young and old carbon pools throughout Cambisol and Luvisol profiles under forests. Soil Biology and Biochemistry. 2006;38(8):2411–24.

[34] Zech W, Senesi N, Guggenberger G, Kaiser K, Lehmann J, Miano TM, et al Factors controlling humification and mineralization of soil organic matter in the tropics. Geoderma. 1997;79(1):117–61.

[35] State Forestry Administration P. R. China (SFAPRC). Statistics of Forest Resources in China (1999–2003). 2005.(in Chinese).

[36] Xu XJ, Du HQ, Zhou GM, Ge HL, Shi YJ, Zhou YF, et al Estimation of aboveground carbon stock of Moso bamboo (Phyllostachys heterocycla var. pubescens) forest with a Landsat Thematic Mapper image. International journal of remote sensing. 2011;32(5):1431–48.

[37] Wang B, Wei WJ, Liu CJ, You WZ, Niu X, Man RZ. Biomass and carbon stock in Moso bamboo forests in subtropical China: characteristics and implications. Journal of Tropical Forest Science. 2013;25(1):137–48.

[38] Zhou GM, Jiang PK. Density, storage and spatial distribution of carbon in Phyllostachy pubescens forest. Scientia Silvae Sinicae. 2004;40(6):20–4.(in Chinese).

[39] State Soil Survey Service of China. Soils of China. Beijing: Agricultural Press 1998.(in Chinese).

[40] World Reference Base for Soil Resources (WRB). A framework for international classification, correlation and communication. World soil resources reports 2006;103.

[41] Zhang HX, Zhuang SY, Ji HB, Zhou S, Sun B. Estimating Carbon Storage of Moso Bamboo Forest Ecosystem in Southern China. Soils. 2014;46(3):413–8.(in Chinese).

[42] Guo LB, Gifford RM. Soil carbon stocks and land use change: a meta analysis. Global change biology. 2002;8(4):345–60.

[43] Zhang CH. Advance of Application of Spectroscopy in Humic Substance. Chinese Journal of Spectroscopy Laboratory. 2011;28(2):693–6.(in Chinese).

[44] Kononova MM. Soil organic matter: Its nature, its role in soil formation and in soil fertility. Oxford: Pergamon 1966.

[45] Stevenson IL, Schnitzer M. Transmission electron microscopy of extracted fulvic and humic acids. Soil Science. 1982;133(3):179–85.

[46] Chin YP, Aiken GR, Danielsen KM. Binding of pyrene to aquatic and commercial humic substances: the role of molecular weight and aromaticity. Environmental Science & Technology. 1997;31(6):1630–5.

[47] Uyguner CS, Hellriegel C, Otto W, Larive CK. Characterization of humic substances: Implications for trihalomethane formation. Analytical and bioanalytical chemistry. 2004;378(6):1579–86. 15214420 10.1007/s00216-003-2451-7

[48] Mathers NJ, Xu ZH, Berners-Price SJ, Senake Perera MC, Saffigna PG. Hydrofluoric acid pre-treatment for improving 13C CPMAS NMR spectral quality of forest soils in south-east Queensland, Australia. Soil Research. 2002;40(4):665–74.

[49] Zhang T, Li YF, Chang SX, Jiang PK, Zhou GM, Liu J, et al Converting paddy fields to Lei bamboo (Phyllostachys praecox) stands affected soil nutrient concentrations, labile organic carbon pools, and organic carbon chemical compositions. Plant and soil. 2013;367(1–2):249–61.

[50] Baidock JA, Oades JM, Nelson PN, Skene TM, Golchin A, Clarke P. Assessing the extent of decomposition of natural organic materials using solid-state C-13 NMR spectroscopy. Aust J Soil Res. 1997;35(5):1061–83.

[51] Dai KOH, Johnson CE, Driscoll CT. Organic matter chemistry and dynamics in clear-cut and unmanaged hardwood forest ecosystems. Biogeochemistry. 2001;54(1):51–83.

[52] Smith JL, Halvorson JJ, Bolton HJ. Soil properties and microbial activity across a 500m elevation gradient in a semi-arid environment. Soil Biology and Biochemistry. 2002;34(11):1749–57.

[53] Dahlgren RA, Boettinger JL, Huntington GL, Amundson RG. Soil development along an elevational transect in the western Sierra Nevada, California. Geoderma. 1997;78(3):207–36.

[54] Garten CT Jr. Potential net soil N mineralization and decomposition of glycine-13 C in forest soils along an elevation gradient. Soil Biology and Biochemistry. 2004;36(9):1491–6.

[55] Bonito GM, Coleman DC, Haines BL, Cabrera ML. Can nitrogen budgets explain differences in soil nitrogen mineralization rates of forest stands along an elevation gradient? Forest Ecology and Management. 2003;176(1):563–74.

[56] Coûteaux MM, Sarmiento L, Bottner P, Acevedo D, Thiéry JM. Decomposition of standard plant material along an altitudinal transect (65–3968m) in the tropical Andes. Soil Biology and Biochemistry. 2002;34(1):69–78.

[57] Niklińska M, Klimek B. Effect of temperature on the respiration rate of forest soil organic layer along an elevation gradient in the Polish Carpathians. Biology and Fertility of Soils. 2007;43(5):511–8.

[58] Du BM, Kang HZ, Pumpanen J, Zhu PH, Yin S, Zou Q, et al Soil organic carbon stock and chemical composition along an altitude gradient in the Lushan Mountain, subtropical China. Ecol Res. 2014;29(3):433–9. 10.1007/s11284-014-1135-4

[59] Zhu B, Wang XP, Fang JY, Piao SL, Shen HH, Zhao SQ, et al Altitudinal changes in carbon storage of temperate forests on Mt Changbai, Northeast China. Journal of plant research. 2010;123(4):439–52. 10.1007/s10265-009-0301-1 20127501

[60] Tewksbury CE, Van Miegroet H. Soil organic carbon dynamics along a climatic gradient in a southern Appalachian spruce-fir forest. Canadian journal of forest research. 2007;37(7):1161–72.

[61] Zhang M, Zhang XK, Liang WJ, Jiang Y, Dai GH, Wang XG, et al Distribution of soil organic carbon fractions along the altitudinal gradient in Changbai Mountain, China. Pedosphere. 2011;21(5):615–20.

[62] Charan G, Bharti VK, Jadhav SE, Kumar S, Angchok D, Acharya S, et al Altitudinal variations in soil carbon storage and distribution patterns in cold desert high altitude microclimate of India. African Journal of Agricultural Research. 2012;7(47):6313–9.

[63] Gupta MK, Sharma SD. Sequestered organic carbon status in the soils under grassland in Uttarakhand State, India. Applied Ecology and Environmental Sciences. 2013;1(1):7–9.

[64] Wei YW, Li MH, Chen H, Lewis BJ, Yu DP, Zhou L, et al Variation in carbon storage and its distribution by stand age and forest type in boreal and temperate forests in northeastern China. Plos One. 2013;8(8):1–9. 10.1371/journal.pone.0072201 PMC3748074 23977252

[65] Huang CY, Jien SH, Chen TH, Tian Gl, Chiu CY. Soluble organic C and N and their relationships with soil organic C and N and microbial characteristics in moso bamboo (*Phyllostachys edulis*) plantations along an elevation gradient in Central Taiwan. Journal of Soils and Sediments. 2014;14(6):1061–70. 10.1007/s11368-014-0870-z

[66] Garten CT Jr, Post WM III, Hanson PJ, Cooper LW. Forest soil carbon inventories and dynamics along an elevation gradient in the southern Appalachian Mountains. Biogeochemistry. 1999;45(2):115–45.

[67] Kirschbaum MUF. Will changes in soil organic carbon act as a positive or negative feedback on global warming? Biogeochemistry. 2000;48(1):21–51.

[68] Matus F, Garrido E, Sepúlveda N, Cárcamo I, Panichini M, Zagal E. Relationship between extractable Al and organic C in volcanic soils of Chile. Geoderma. 2008;148(2):180–8. 10.1016/j.geoderma.2008.10.004

[69] Rasmussen C, Torn MS, Southard RJ. Mineral Assemblage and Aggregates Control Carbon Dynamics in a California Conifer Forest. Soil Science Society of America Journal. 2005;69(6):1711 10.2136/sssaj2005.0040

[70] Ma L, Yang LZ, Ci E, Wang Y, Yin SX, Shen MX. Humus composition and stable carbon isotope natural abundance in paddy soil under long-term fertilization. Chinese Journal of Applied Ecology. 2008;19(9):1951–8.(in Chinese). 19102308

[71] Liu SX, Liu JS, Zhao LP. A study on the composition characters of combined humus in cultivated soils of jilin province. Journal of Jilin Agricultural University 2002;24(1):72–6.(in Chinese).

[72] Jien SH, Chen TH, Chiu CY. Effects of afforestation on soil organic matter characteristics under subtropical forests with low elevation. Journal of Forest Research. 2011;16(4):275–83. 10.1007/s10310-010-0231-8

[73] Mathers NJ, Xu ZH. Solid-state 13 C NMR spectroscopy: characterization of soil organic matter under two contrasting residue management regimes in a 2-year-old pine plantation of subtropical Australia. Geoderma. 2003;114(1):19–31.

[74] Li YF, Jiang PK, Chang SX, Wu JS, Lin L. Organic mulch and fertilization affect soil carbon pools and forms under intensively managed bamboo (Phyllostachys praecox) forests in southeast China. Journal of Soils and Sediments. 2010;10(4):739–47.

[75] Spielvogel S, Prietzel J, Kögel-Knabner I. Soil organic matter stabilization in acidic forest soils is preferential and soil type-specific. European Journal of Soil Science. 2008;59(4):674–92.

[76] Rumpel C, Chabbi A. Response of bulk chemical composition, lignin and carbohydrate signature to grassland conversion in a ley-arable cropping system. Nutrient cycling in agroecosystems. 2010;88(2):173–82.

[77] Martin-Neto L, Rosell R, Sposito G. Correlation of spectroscopic indicators of humification with mean annual rainfall along a temperate grassland climosequence. Geoderma. 1998;81(3):305–11.

[78] Senesi N, D'orazio V, Ricca G. Humic acids in the first generation of EUROSOILS. Geoderma. 2003;116(3):325–44.

[79] Trumbore SE, Chadwick OA, Amundson R. Rapid exchange between soil carbon and atmospheric carbon dioxide driven by temperature change. Science. 1996;272:393–6.

[80] MacDonald NW, Randlett DL, Zak DR. Soil warming and carbon loss from a lake states spodosol. Soil Science Society of America Journal. 1999;63(1):211–8.

[81] Kirschbaum MUF. The temperature dependence of soil organic matter decomposition, and the effect of global warming on soil organic C storage. Soil Biology and biochemistry. 1995;27(6):753–60.

[82] Raich JW, Potter CS. Global patterns of carbon dioxide emissions from soils. Global Biogeochemical Cycles. 1995;9(1):23–36.

