# Supplementary material for: Soil Organic Carbon Pool and Its Chemical Composition in Phyllostachy pubescens Forests at Two Altitudes in Jian-ou City, China
Source: PLoS One. 2015 Dec 30;10(12):e0146029. doi: 10.1371/journal.pone.0146029 (PMC4696818; doi:10.1371/journal.pone.0146029)
Supplement: S1 Table — LAS: low-altitude site; HAS: high-altitude site. (DOC) [file pone.0146029.s001.doc]

| **Soil profile** | **Elevation** | **NO_3_^−^–N**  **(mg kg^−1^)** |
| --- | --- | --- |
| **0–10 cm** | **LAS** | 31.49a |
|  | **HAS** | 17.27a |
| **10–20 cm** | **LAS** | 23.32a |
|  | **HAS** | 13.14a |
| **20–40 cm** | **LAS** | 13.16a |
|  | **HAS** | 10.56a |
| **40–60 cm** | **LAS** | 8.94a |
|  | **HAS** | 6.63a |
